# Predictive value of the cow’s milk skin prick test in infantile colic

**DOI:** 10.4103/0256-4947.72269

**Published:** 2010

**Authors:** Hossein Moravej, Mohammad H. Imanieh, Sara Kashef, Farhad Handjani, Fardin Eghterdari

**Affiliations:** a From the Department of Pediatrics, Shiraz University of Medical Sciences, Shiraz, Iran; b From the Allergy Research Center, Shiraz University of Medical Sciences, Shiraz, Iran

## Abstract

**BACKGROUND AND OBJECTIVES:**

Infantile colic is a common problem among young infants. Cow’s milk allergy has been suggested as one of the causes. We aimed to investigate the value of the cow’s milk skin test for the diagnosis of cow’s milk allergy in exclusively breast-fed infants with infantile colic.

**METHODS:**

Exclusively breast-fed infants with infantile colic were enrolled in this study. On the first visit, the average hours of crying of the infant in a 24-h period were recorded and the cow’s milk skin test was performed. If the infant had a positive skin test, elimination of cow’s milk from the mothers’ diet was advised. Infants with negative skin tests were divided into case and control groups. Cow’s milk was eliminated from the diet of mothers in the case group. After 2 weeks, the number of hours of crying were recorded again. The reduction in the crying hours was compared between the two groups using the chi-square test.

**RESULTS:**

Skin tests were positive in 3 of 114 cases (2.6%) of infantile colic. All three cases recovered completely following elimination of cow’s milk from the mother’s diet. Among the 111 patients with negative skin tests, 77 patients completed the study: 35 in the case group and 42 in the control group. The reduction in crying hours in infants in the case group was not significantly different from that in the control group.

**CONCLUSION:**

Elimination of cow’s milk from the mothers’ diet is not beneficial for infants with a negative skin test. Infants with a positive skin test may benefit from this management.

Infantile colic affects up to 28% of infants in the first few months of life.^1^,^2^ Frequent episodes of crying in these infants may cause significant disruption of family interactions.^3^–^6^ Food allergy is a common problem in infants and small children. The classical allergist’s tool for diagnosing allergies is skin prick testing (SPT), and this can also be applied for diagnosis of food allergy.^7^ Despite the availability of various skin and laboratory tests, the current clinically valid method to demonstrate significant food allergy is elimination and challenge with the suspected food.^8^ Cow’s milk protein is the most common food allergen in infants under 1 year of age. Due to an increasing number of infants in need of diagnostic workup, it has become acceptable to diagnose cow’s milk allergy in infants on the basis of a positive SPT and cessation of symptoms on an elimination diet.^9^

The role of cow’s milk allergy in infantile colic is controversial.^10^ Some studies have reported a reduction in persistent crying after elimination of cow’s milk and other food proteins from the mother’s diet,^11^,^12^ but other studies have reported that maternal diet does not seem to be related to infantile colic in breast-fed infants.^13^,^14^ In the present study, we aimed to investigate the value of SPT in the diagnosis of cow’s milk allergy in patients with infantile colic.

## METHODS

This single-blind randomized clinical trial was done on exclusively breast-fed infants with history of infantile colic. All suspected cases of infantile colic between the ages of 3 weeks and 3 months were seen by a pediatrician, who diagnosed infantile colic on the basis of a complete history and thorough physical examination. Other probable etiologies were ruled out. Diagnosis of infantile colic was made on the basis of Wessel criteria, i.e., severe crying in infants between 3 weeks and 3 months old, occurring at least 3 days per week and lasting 3 hours per day.^15^

For each infant, the parent(s) were asked about the average number of hours of crying in 24 hours and this was recorded. After explaining the nature of the study to the parents and obtaining written informed consent, SPT was performed for all the infants. SPT was done on the volar aspect of the forearm by an expert nurse, under the close supervision of a pediatric allergist, with cow’s milk extract (Allergopharma, Germany), fresh cow’s milk, histamine dihydrochloride (as a positive control), and glycerol (as a negative control). All necessary precautions for management of any possible adverse reaction were taken. Reactions were read after 15 minutes, and a response was considered positive if the mean diameter of the wheal was at least 3 mm more than the negative control.

Infants with a positive SPT were excluded. The rest were divided randomly into case and control groups. For infants in the control group, no change in the mother’s diet was suggested and the parents were instructed to bring their babies for follow-up 2 weeks later. Mothers of infants in the case group were asked to avoid cow and goat milk as well as dairy products for 2 weeks. These mothers were prescribed calcium supplements and were instructed to take a calcium-rich diet. The infants in the case group were also seen again at follow-up 2 weeks later.

On the second visit, the same parents were asked about the average number of hours of crying per day, and the infants were reexamined by a pediatrician who did not know the group allocation of the infants. Those infants whose crying hours were decreased by 1–2 hours per day were labeled as partial responders, and decrease in crying hours by more than 2 hours per day was considered a complete response. No difference in number of crying hours or a decrease of less than 1 hour per day was labeled as no response. Infants whose parents did not follow all of the instructions and those who were not brought for follow-up after the first visit were excluded from the study. The responses of the case and control groups were compared using the chi-square test. *P*=.05 indicated statistical significance.

## RESULTS

**Figure 1:**
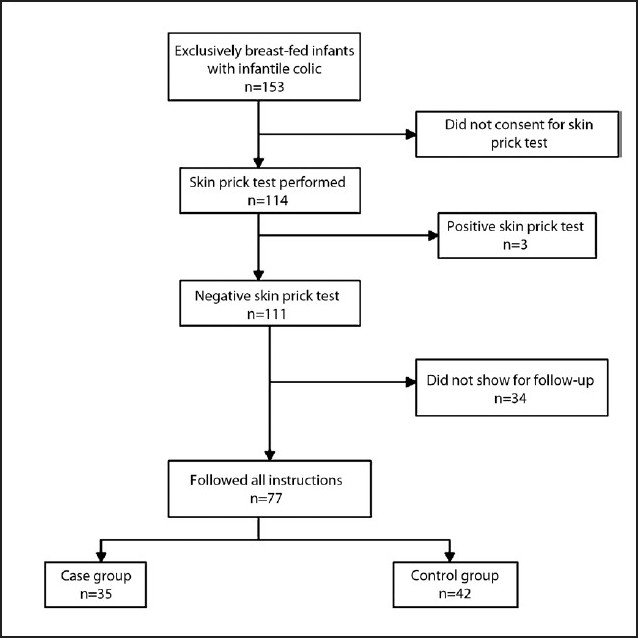
Stages of the study.

Of 153 exclusively breast-fed infants with colic, the parents of 114 cases allowed us to do the SPT. Among these infants, three cases had a positive SPT (2.6%). For these three cases, elimination of cow’s milk products from the maternal diet resulted in complete recovery. The 111 SPT-negative cases were randomly divided into case and control groups. Of these infants, 35 infants of the case group (mothers asked to avoid cow and goat milk and other dairy products) and 42 infants of the control group (regular diet) returned for follow-up after 2 weeks. The different stages of the study are detailed in **Figure 1**. The change in the number of crying hours in the case and control groups were compared using the chi-square test, but no statistically significant difference was seen between the two groups (**Table 1**).

**Table 1:** Comparison of response rate between case and control groups.

Response	Case	Control
Complete response	14	11
Partial response	10	17
No response	11	14

**Total**	**35**	**42**

(Chi-square=1.19; *P*=.38)

## DISCUSSION

In this study, the effect of cow’s milk allergy was evaluated in infantile colic through two methods: elimination diet and SPT. SPT with cow’s milk was performed for 114 cases with infantile colic and was positive in 2.6% of cases. Previous studies have demonstrated that SPT has a high specificity and low sensitivity for immediate-type food reactions in infants under the age of 1 year.^9^ Indeed, a positive test in these infants suggests a relevant food allergy.^16^ To the best of our knowledge, there are no previous studies of SPT in patients with infantile colic.

In this study the elimination of cow’s milk and dairy products from the diet of mothers of exclusively breast-fed infants with negative SPT had no effect on the attacks of colic. The effect of a low-allergen diet on infantile colic has been studied in multiple studies and has shown different results. Hill et al studied the effect of a low-allergen diet on colic among breast-fed infants.^17^ They instructed mothers to exclude multiple nutrients from their diets for a duration of 1 week and found that a low-allergen maternal diet is associated with a significant reduction in the time the infant spends in crying/fussing. It is clear that elimination diets have associated risks, particularly if sustained for long periods,^18^ and elimination of multiple nutrients may not be a proper regimen for management in all cases.

Evans and coworkers^13^ performed a similar study and concluded that maternal diet does not affect infantile colic in breast-fed infants. Taubman^19^ compared parental counseling with elimination of cow’s milk from the diet of infants and concluded that parental counseling is more effective than elimination of cow’s milk for treatment of infantile colic. Garrison and Christakis^14^ conducted a systematic review of the management of infantile colic and concluded that there is no conclusive evidence of the efficacy of a low-allergen diet in the management of infantile colic in exclusively breast-fed infants and that more studies are needed.We found that elimination of cow’s milk from the mothers’ diet has no effects on the symptoms of colic in infants with a negative SPT. Infants with positive SPT may benefit from elimination of cow’s milk products from the maternal diet. More studies are needed to determine which patient will benefit from an elimination diet.

Our study had several limitations. There was a high dropout rate and the number of positive SPTs was low. Because of the nature of the intervention, the study could not be conducted as a double-blind trial. Furthermore, although the gold standard method for detection of food allergy is a double-blind placebo-controlled challenge test, because of the self-limiting nature of colic, the challenge test was not conducted in these infants.
